# Editorial: Computational modeling methods for naturalistic neuroimaging data

**DOI:** 10.3389/fncom.2023.1117945

**Published:** 2023-01-18

**Authors:** Yudan Ren, Huan Liu, Shu Zhang, Jinglei Lv

**Affiliations:** ^1^School of Information Science and Technology, Northwest University, Xi'an, China; ^2^School of Computer Science and Communication Engineering, Jiangsu University, Zhenjiang, Jiangsu, China; ^3^Department of Computer Science, Center for Brain and Brain-Inspired Computing Research, Northwestern Polytechnical University, Xi'an, China; ^4^School of Biomedical Engineering and Brain and Mind Center, University of Sydney, Sydney, NSW, Australia

**Keywords:** naturalistic paradigm, neuroimaging, deep learning, machine learning, brain disorder

Over the past decade, there is increasing interest in understanding the human brain function in real-life scenarios. Therefore, efforts have been put in developing neuroimaging studies with ecologically-valid naturalistic paradigms, such as video watching. We believe such approaches are promising to provide tremendous new knowledge for neuroscience (Lv et al., 2017; Ren et al., 2018; Zhao et al., 2018) and mental health (Ren et al., 2017). However, to fully mine the rich information in the naturalistic neuroimaging data, computational modeling innovation is highly demanded. Thus, the aims of this Research Topic are to promote the state-of-the-art understanding of neural processes driven by naturalistic stimuli, showcase advanced methods, and ultimately envision potential naturalistic neuroimaging biomarkers in mental health conditions. Authors representing a broad spectrum of interdisciplinary research fields, e.g., neuroscience and computer science, have contributed methodological advances, empirical findings, and applications for brain disorder research.

## Naturalistic neuroimaging

Naturalistic paradigms can be referred to those designs that adopt the dynamic and multimodal stimuli that can largely approximate real-life experience. Generally, there are two categories, namely perceptually-oriented paradigms and dynamic interactions with the paradigms. Most of studies in this Research Topic employ the perceptually-oriented paradigms that can be defined as passively viewing/listening of rich dynamic and multimodal stimuli, such as video clips (Le et al.; Hu, Zhang et al.), reading science fictions/social media posts (Hu, Cui et al.), and movies (Ren et al.; Chen et al.; Ou et al.). Moreover, one study focuses on visual perception process by employing a continuous visual searching design (Kiefer et al.). Intriguingly, diverse modalities of neuroimaging have been adopted, including functional magnetic resonance imaging (fMRI), Magnetoencephalography (MEG) with eye tracking data, and electroencephalogram (EEG). With relatively higher test-retest reliability (Wang et al., 2017) and less microsleeps or head movements, naturalistic paradigms can provide a promising condition for studying brain disorders.

## Computational modeling methods for naturalistic neuroimaging

Among the studies, several authors have proposed statistics-based frameworks for naturalistic neuroimaging analyses. To investigate functional networks related to goal-directed visual processing driven by active visual stimuli, Kiefer et al. applied the spatiotemporal cluster permutation testing and generalized partial directed coherence to detect the region of interest and to reveal the directed connectivity among them. Posterior insula, transverse temporal gyri, superior temporal gyrus, and supramarginal gyrus formed a highly connected network during guided visual searching, with supramarginal gyrus acting as a central part for this process. In another work, Ou et al. combined sliding time windows with inter-Subject functional correlation method (ISFC) to detect movie events evoked fMRI activity, which shows significant group differences in ISFC patterns between brains with autism spectrum disorder (ASD) and typical development.

Machine learning has also become a powerful tool. Combining independent component analysis (ICA) and dynamic function network connectivity analysis, Hu, Cui et al. explored the cerebral carryover effects of natural reading tasks (including reading social media posts and science fiction) on brain dynamics. The study found that reading science fiction could substantially increase brain activity and network efficiency, while social media was related to abnormal functional connectivity among default mode network, visual network, and frontoparietal network. As EEG microstate analysis is an effective means to study spatial and temporal dynamics of brain, Hu, Zhang et al. answered the question of whether the topographical clustering strategies affect the performance of microstate detection in naturalistic EEG microstate analysis. The study systematically compared the performance in terms of microstate quality, task efficacy, and computational efficiency, and found that the single-trial-based topographical clustering could offer a good choice for neural activity study on naturalistic EEG data.

One half of the studies proposed or applied deep learning methods. To decode the continuous task states, Wu et al. proposed a framework combining convolutional neural network and continuous wavelet transform to process the temporal dynamics of single-voxel fMRI time-series, which provides novel insight to precise localization of abnormal brain activity. Ren et al. proposed a novel two-stage deep belief network with neural architecture search (DBN-NAS) framework to examine both group-level and individual-level functional brain networks and their naturalistic fMRI activities, where the proposed model can not only automatically determine the optimal neural architecture, but also can effectively reveal the hierarchical organization of brain function under naturalistic paradigm. To reconstruct complex and dynamic visual perception from naturalistic brain activity, Le et al. applied an image-to-image transformation network, where brain activations in visual cortices were first embedded as visual representations *via* retinotopic mapping and these visual representations were then transformed into the original naturalistic images and videos. This type of decoding study is critical for understanding the functional mechanism of human brain, and especially benefits brain-computer interface. Finally, to tackle variability across subjects and sessions in emotion recognition task, Chen et al. took marginal distribution of EEG data into account and proposed the multi-source marginal distribution adaptation (MS-MDA) for cross-subject and cross-session EEG emotion recognition, consequently achieving the state-of-the-art performance in transfer learning scenarios.

## Conclusion

These studies have made distinguished contributions to the field of naturalistic neuroimaging. Most of the studies are pioneer research for method development, but we expect these methods to make a broader impact on the brain disorder analysis using naturalistic paradigm. We thank not only the authors for their excellent contributions but also the reviewers for sharing their expertise. Their efforts have ensured this Research Topic as a collection of high-quality articles.

## Author contributions

YR wrote the editorial. HL, SZ, and JL edited the editorial. All authors contributed to the article and approved the submitted version.
